# Spin revolution breaks time reversal symmetry of rolling magnets

**DOI:** 10.1038/s41598-022-17766-z

**Published:** 2022-08-10

**Authors:** Elena Y. Vedmedenko, Roland Wiesendanger

**Affiliations:** Physics Department, University of Hamburg, Jungiusstr 11, 20355 Hamburg, Germany

**Keywords:** Applied physics, Physics, Magnetic properties and materials

## Abstract

The classical laws of physics are usually invariant under time reversal. Here, we reveal a novel class of magnetomechanical effects rigorously breaking time-reversal symmetry. These effects are based on the mechanical rotation of a hard magnet around its magnetization axis in the presence of friction and an external magnetic field, which we call spin revolution. The spin revolution leads to a variety of symmetry breaking phenomena including upward propulsion on vertical surfaces defying gravity as well as magnetic gyroscopic motion that is perpendicular to the applied force. The angular momentum of spin revolution differs from those of the magnetic field, the magnetic torque, the rolling axis, and the net torque about the rolling axis. The spin revolution emerges spontaneously, without external rotations, and offers various applications in areas such as magnetism, robotics and energy harvesting.

## Introduction

**Figure 1:**
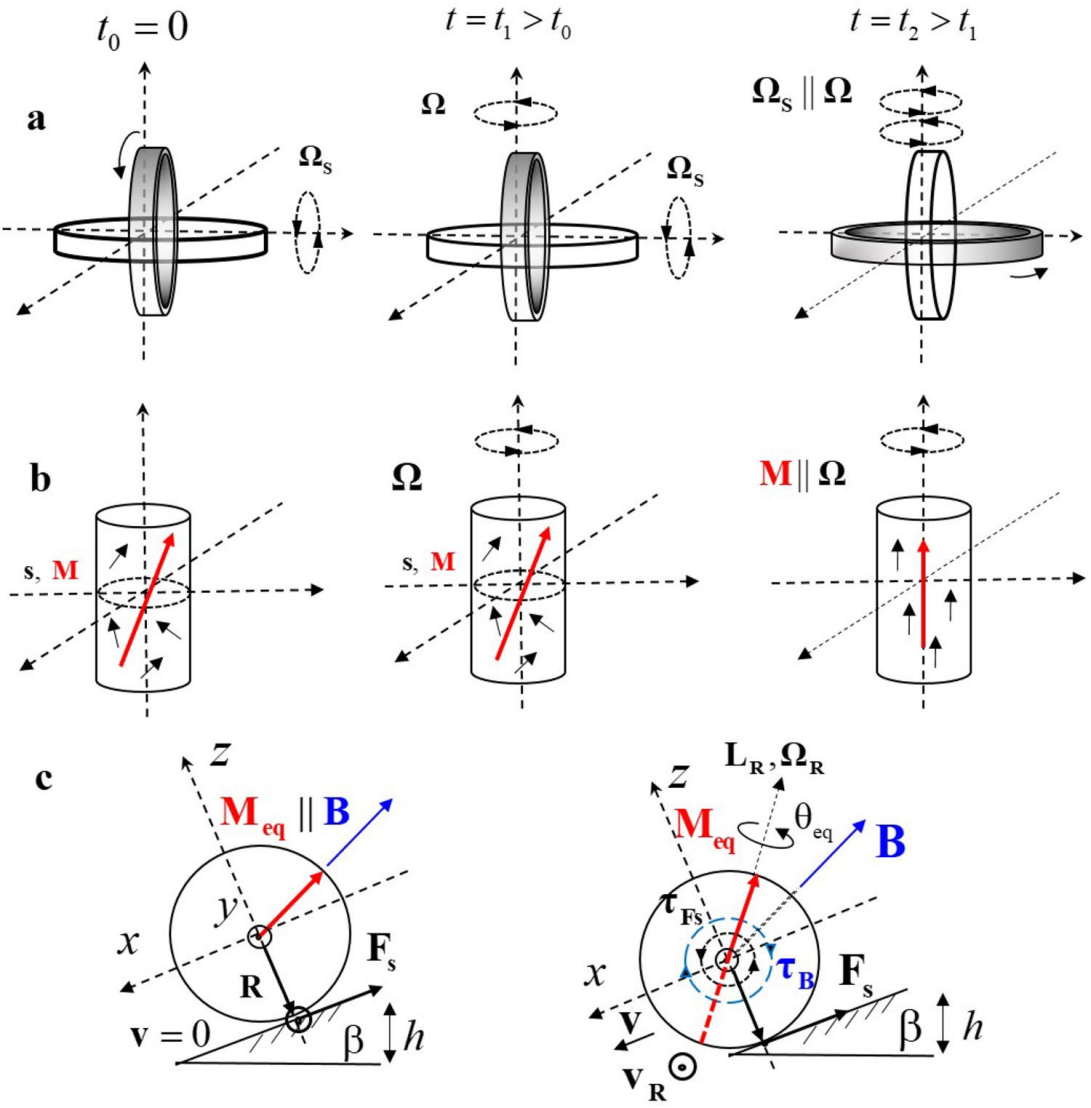
Gyroscopic, gyromagnetic and spin revolution effects. (**a**) Schematic representation of a mechanical gyroscope, which can be controlled by an external rotation ${{\varvec{\Omega}}}$ to align the spinning axis ${{\varvec{\Omega}}}_{{\text{s}}}$ with ${{\varvec{\Omega}}}$. (**b**) Schematic representation of a gyromagnetic effect, in which the magnetization can be controlled by external rotation ${{\varvec{\Omega}}}$ to align the magnetization **M** with ${{\varvec{\Omega}}}$. (**c**) Schematic side view of a spin revolution effect. Initially, the magnetized sphere is at rest and ${\mathbf{M||B}}$. When the sphere starts to roll down an incline, the magnetization departs from its initial orientation, relaxes to a direction ensuring minimal total torque about rolling axis ${\mathcal{T}}_{{{\text{c.m.}}}} \to \min$, and starts to revolve with ${{\varvec{\Omega}}}_{{\text{R}}}$. Vectors are represented by bold letters for clarity. Reprinted with permission from^11^.

Symmetry breaking leads to fascinating effects across sciences, from the appearance of spontaneous magnetization to exciting properties of two-dimensional layered material systems^1,2^. Here, we reveal a novel magnetomechanical effect rigorously breaking time-reversal symmetry. State-of-the-art *gyroscopic* effects involve the motion of spinning objects. A spinning axis can be defined by its mechanical angular momentum ${\mathbf{L}}_{{\text{s}}}$ and velocity ${{\varvec{\Omega}}}_{{\text{s}}}$. A spinning object can be controlled or manipulated by another external rotation with angular velocity ${{\varvec{\Omega}}}$ to align ${{\varvec{\Omega}}}_{{\text{s}}}$ and ${{\varvec{\Omega}}}$ due to the Coriolis force as shown in Fig. 1a^3^. State-of-the-art *gyromagnetic* effects are based on the motion of spinning magnetic objects. In this case, the magnetization **M** stemming from the spin angular momentum can be controlled or manipulated by an external rotation ${{\varvec{\Omega}}}$ to align **M** and ${{\varvec{\Omega}}}$ via the spin-rotation coupling as shown in Fig. 1b^4–7^. In all these cases, an object subject to manipulation is initially spinning around a well-defined axis ${{\varvec{\Omega}}}_{{\text{s}}}$ in the laboratory frame.

The magnetomechanical effect introduced here concerns a hard magnetic object (conducting or insulating) with magnetization **M** that does not spin initially, but rather rests at a particular position (see Fig. 1c). Several torques, including a gravitational and a magnetic torque, are acting on the object and we are interested in the characteristics of the resulting movement (e.g. rolling) of such an object (e.g. a sphere). In a first approximation, we neglect all effects of moving electric charges or electric fields due to the time-dependent magnetization because of their weakness. Furthermore, we are interested in the regime of rolling without slipping. Our analysis shows that, when a net torque about an object’s rolling axis is minimized ${\mathbf{\rm T}}_{{\text{c.m.}}} = \sum_{i = 1}^{N} {{{\varvec{\uptau}}}_{i} } \to \min$, the object spins up with an angular momentum **L**_R_, pointing in a direction which differs from those of the magnetic field, the magnetic torque, the rolling axis, and the net torque about the rolling axis, and starts to move perpendicularly to an applied force with a velocity **v**_R_. We denote this combination of spontaneous rotation and translational movement as spin revolution (SR). In contrast to known effects, the SR emerges spontaneously, without application of any external rotation about **L**_R_. In contrast to the electron spin, which is antiparallel to its magnetic moment, **L**_R_ can be parallel or antiparallel to an equilibrium magnetization orientation **M**_eq_. The key ingredient for this counterintuitive motion is the minimization of the total torque about the rolling axis leading to the emergence of torque about **M**_eq_. If **M**_eq_ is inclined with respect to the surface’s normal, the subsequent motion corresponds to the rolling with inclined axis. This motion breaks the time-reversal symmetry of the moving magnet and leads to circular trajectories as well as to vertical propulsion defying gravity. The time-reversal symmetry breaking paves the way for an effective interconversion of translational and rotational motion and, by that means, to numerous applications in mechanics, robotics, energy harvesting and magnetism. For example, SR allows the development of rope-less, rail-less and hydraulics-less elevators, linear motors or angle gears. It can also be applied to the controlled rotational and translational motion of magnetic particles.

## Results

### Overview of experiments

To make the objectives of the manuscript as clear as possible, we start with a short overview of our experiments and then present a detailed quantitative analysis. The first simplest experiment serves to clarify the question: what is the difference between the rolling motion of non-magnetic and hard-magnetic bodies? In this experiment, magnetized NdFeB spheres and their non-magnetic metallic counterparts of identical diameters were let to roll down an incline or to roll on a plane due to an external force ${\mathbf{F}}_{{{\text{appl}}}}$ (see description and Movie S1–S4^8^). Non-magnetic spheres on an incline were rolling as expected linearly, rotating about a standard horizontal axis, while a magnetic sphere revolved; that is, it spun around an almost vertical axis and at the same time was on a non-linear trajectory just like a charge under the action of the Lorentz force or a planet on its orbit. To clearly show this spinning we have restricted the motion of a magnetic sphere to one dimension using a transparent test tube in Movie S1–S2^8^. In Movie S3^8^ one can clearly appreciate a Lorentz-like force acting on a magnetic sphere, while this force is absent for a non-magnetic sphere. The almost vertical orientation of the rolling axis is counterintuitive because it coincides neither with that of the earth’ magnetic field, nor with the horizontal mechanical rolling axis, nor with the vectorial sum of the corresponding torques. The Lorentz-like force shown in Movie S3^8^ is interesting because it leads to a non-local breaking of time-reversal symmetry. That is, if one reverses the magnetic field orientation, the Lorentz force reverses, while the Lorentz-like force in our case will not, as explained in detail in^8^. This non-local symmetry breaking becomes particularly pronounced in the experiment with two magnetic spheres, which were put each into a vertical non-magnetic tube. The tubes were placed close to one another and the spheres arranged themselves on the internal sides of the tubes due to the magnetic attraction ${\mathbf{F}}_{{\text{m}}}^{12}$ and ${\mathbf{F}}_{{\text{m}}}^{21}$^10^ as shown in Movie S5^8^. When the tubes were rotated due to ${\mathbf{F}}_{{{\text{appl}}}}$ about their vertical axes, the spheres moved always upwards independently of the sense of rotation and of the magnetization orientation of the spheres. This is in contrast to the behavior of time-reversal invariant systems. For instance, turning a screwdriver clockwise allows one to tighten, while turning it counterclockwise to loosen a screw, while neither the change of the tube’s rotation nor the change of the sphere’s magnetization orientation will change the direction of sphere’s translation.

### Equations of motion

To illustrate the nature of the effect in a more detailed way, we start with a standard problem in rotational kinematics considering a sphere rolling down an inclined plane. A rolling motion can be represented as a combination of a rotation about a rolling axis and its translation. A rotational torque arises from an instantaneous static friction force **F**_s_ and equals ${{\varvec{\uptau}}}_{{{\text{Fs}}}} = {\mathbf{R}} \times {\mathbf{F}}_{{\text{s}}}$ with **R** being the vector connecting the center-of-mass (c.m.) and a contact point. In the coordinate system connected with the contact point (see Fig. 1c) the rolling axis coincides with ${{\varvec{\uptau}}}_{{{\text{Fs}}}} = (0,\frac{2}{7}mgR {{\sin}}\beta ,0) = (0,\tau_{{{\text{Fs}}}}^{{\text{y}}} ,0)$, with $\beta$ being the inclination angle (see^8^, part A), *m* the mass and *g* the gravitational acceleration. It is well known that a homogeneously magnetized solid sphere is equivalent to a point dipole placed at its center^9,10^. So, if a sphere is magnetized, a uniform magnetic field **B** (e.g. the earth’s field) exerts a torque ${{\varvec{\uptau}}}_{{\text{B}}} = {\mathbf{M}}(t) \times {\mathbf{B}} = M_{{\text{s}}} {\mathbf{e}}_{{\text{M}}} (t) \times {\mathbf{B}}$ on this dipole (with *M*_s_ being the saturation magnetization and ${\mathbf{e}}_{{\text{M}}} (t)$ the unit magnetization vector), and the net mechanical torque becomes:

1 $${\mathbf{T}}_{{\text{c.m.}}} = \tau_{{{\text{Fs}}}} + \tau_{{\text{B}}} (t) = {\mathbf{R}} \times {\mathbf{F}}_{{\text{s}}} + {\mathbf{M}}(t) \times {\mathbf{B}}$$

We introduce the following equation of motion for the magnetization in the coordinate system of the contact point:

2 $$\partial {\mathbf{M}}(t)/\partial t = - \frac{\gamma \alpha }{{(1 + \alpha^{2} )M_{{\text{s}}} }}{\mathbf{M}}(t) \times ({\mathbf{M}}(t) \times {\mathbf{B}}) - \frac{1}{{(1 + \alpha^{2} )}}{\mathbf{M}}(t) \times {{\varvec{\Omega}}}_{{\text{c.m.}}} ,$$

with the gyromagnetic ratio $\gamma$, the rolling angular velocity ${{\varvec{\Omega}}}_{{\text{c.m.}}}$ and the magnetic damping constant $\alpha$. Here, the first term accounts for the rotation of **M** towards the field due to ${{\varvec{\uptau}}}_{{\text{B}}}$^12,13^, while the second term corresponds to the mechanical precession of **M** around a rolling axis.

The Newton’s equation for the c.m. is:

3 $$m{\mathbf{a}} = m{\mathbf{g}} + {\mathbf{F}}_{{\text{s}}} + {\mathbf{N}}$$

with **N** being the normal force (see^8^, part B). To find **M**(*t*) and, hence, the equilibrium magnetization **M**_eq_ we solve Eqs. (1)–(3).

**Figure 2:**
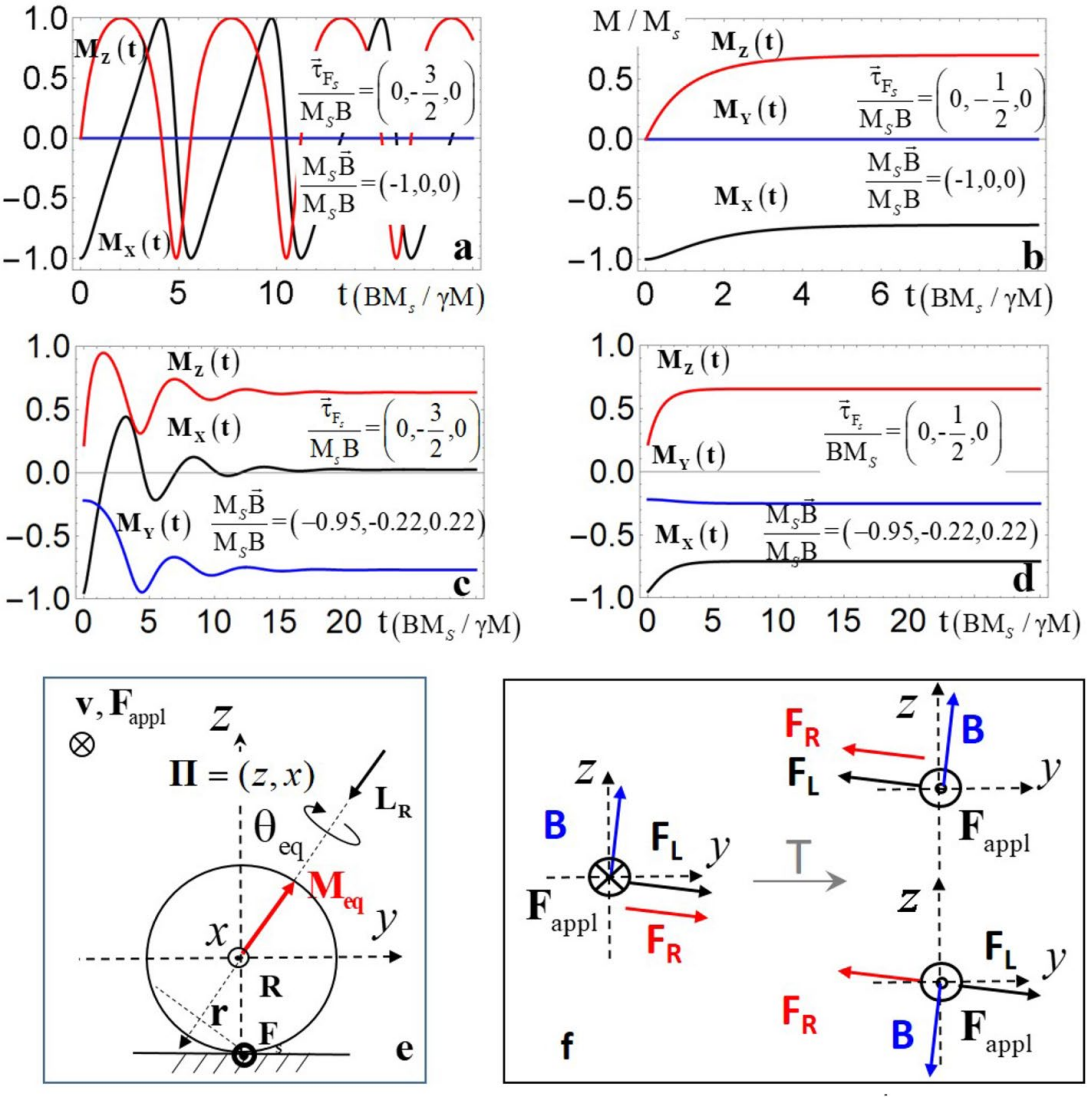
Dynamics of a revolving-up magnet. Initially, the magnetization $\vec{M}$ is parallel to a magnetic field $\vec{B}$. Next, a mechanical torque $\vec{\tau }_{{{\text{F}}s}}$ is applied and $\vec{M}(t)$ evolves for (**a**) $(\vec{v},\vec{B}) \in \Pi = \left( {\hat{z},\hat{x}} \right)$ and $|\vec{\tau }_{{{\text{F}}s}} | > |\vec{\tau }_{{\text{B}}}^{{{{\max}}}} |$; (**b**) $(\vec{v},\vec{B}) \in \Pi$ and $|\vec{\tau }_{{{\text{F}}s}} | \le |\vec{\tau }_{{\text{B}}}^{{{{\max}}}} |$; (**c**) $(\vec{v},\vec{B})\notin\Pi$ and $|\vec{\tau }_{{{\text{F}}s}} | > |\vec{\tau }_{{\text{B}}}^{{{{\max}}}} |$; (**d**) $(\vec{v},\vec{B})\notin\Pi$ and $|\vec{\tau }_{{{\text{F}}s}} | < |\vec{\tau }_{{\text{B}}}^{{{{\max}}}} |$; (**e**) Schematical representation of geometrical axes, forces and angular momentum acting on a rolling magnetic sphere with inclined axis. All definitions correspond to the text; (**f**) Comparison of the Lorentz force for a positive charge and the force due to the SR under the local ($\vec{F}_{{{\text{appl}}}}$ reverses,$\vec{B}$ remains unchanged) and global ($\vec{F}_{{{\text{appl}}}}$ reverses, $\vec{B}$ reverses) time reversal.

First, we solve this set of equations analytically and numerically for **v**, **M**(*t*) and **B** lying in the same plane $(xz) = \Pi$. If the amplitude of the mechanical torque $|{{\varvec{\uptau}}}_{{{\text{Fs}}}} |$ surpasses that of the maximal possible magnetic torque $|{{\varvec{\uptau}}}_{{\text{B}}}^{{{{\max}}}} |$, a sphere starts to roll in a usual way; that is, $\partial {\mathbf{M}}(t)/\partial t \ne 0$ and ${\mathbf{T}}_{{\text{c.m.}}} \ne 0$ as shown in Fig. 2a. In contrast to a usual rolling, however, the sphere rolls non-harmonically due to ${{\varvec{\uptau}}}_{{\text{B}}}$. If $|{{\varvec{\uptau}}}_{{{\text{Fs}}}} | \le |{{\varvec{\uptau}}}_{{\text{B}}}^{{{{\max}}}} |$, both torques may become compensated (${\mathbf{T}}_{{\text{c.m.}}} = 0$) at $\sin [\angle ({\mathbf{M}}_{{{\text{eq}}}} ,{\mathbf{B}})] = \sin [\theta_{{{\text{eq}}}} ] = (RF_{{\text{s}}} )/(M_{{\text{s}}} B)$ (see^8^, part B, Fig. 2b and the right panel of Fig. 1c). The magnetization relaxes towards **M**_eq_ and remains at rest in rotational equilibrium. In the next step we allow for deviations of **v** and **B** from the $\Pi$ plane (see Fig. 2c–e). For $|{{\varvec{\uptau}}}_{{{\text{Fs}}}} | \le |{{\varvec{\uptau}}}_{{\text{B}}}^{{{{\max}}}} |$ the equilibrium magnetization will still relax to a direction minimizing the net torque ${\mathbf{T}}_{{\text{c.m.}}}$, but this orientation will not belong to $\Pi$ anymore as shown in Fig. 2c,d. This, however, means that another torque ${{\varvec{\uptau}}}_{{\text{R}}} = {\mathbf{r}} \times {\mathbf{F}}_{{\text{s}}}$^14^ might emerge if $\left| {{{\varvec{\uptau}}}_{{\text{R}}} } \right| > \left| {{\mathbf{T}}_{{\text{c.m.}}} } \right|$, with **r** being a distance vector pointing from the axis **M**_eq_ to the contact point (see Fig. 2e). Hence, if ${\mathbf{T}}_{{\text{c.m.}}}$ vanishes, that is $\partial {\mathbf{M}}(t)/\partial t \to 0$ (Eq. (2)), the sphere rotates up about **M**_eq_ with angular momentum ${\mathbf{L}}_{{\text{R}}} = I_{{\text{c.m.}}} {{\varvec{\Omega}}}_{{\text{R}}}$ due to ${{\varvec{\uptau}}}_{{\text{R}}}$ ($I_{{\text{c.m.}}}$ is the inertia tensor). This emergent rotation is the essential contribution to the SR.

## Discussion

### Symmetry-breaking motion of a magnet: spin revolution

Any rotation changes the trajectory of an object. Particularly, a spinning object acts as a gyroscope moving perpendicularly to the applied force ${\mathbf{F}}_{{{\text{appl}}}}$ and obeying the dynamics of the gyroscope axis ${{\varvec{\uprho}}}$: $\frac{{\partial {{\varvec{\uprho}}}}}{\partial t} = \frac{{R^{2} }}{{I_{{\text{c.m.}}} \omega }}({\mathbf{L}}_{\rho } \times {\mathbf{F}}_{{{\text{appl}}}} )$^15^. There is, however, an important distinction between a standard gyroscope and a revolving magnet described here: the revolution axis is magnetic. The equation of motion becomes (with $\gamma$ being the gyromagnetic ratio):

4 $$\partial {\mathbf{M}}_{{{\text{eq}}}} /\partial t = \frac{{\gamma R^{2} }}{{I_{{\text{c.m.}}} \Omega_{{\text{R}}} }}({\mathbf{L}}_{{\text{R}}} \times {\mathbf{F}}_{{{\text{appl}}}} )$$

This outcome contains interesting physics. First, a magnetic sphere on an incline should revolve up spontaneously, without any external torque around **M**_eq_ that is different from ${{\varvec{\uptau}}}_{{\text{B}}}$, ${{\varvec{\uptau}}}_{{{\text{Fs}}}}$ as well as their sum. Second, the time-reversal symmetry becomes broken as an action of the time operator *T* on the left side of Eq. (4) $\frac{{T({\mathbf{M}}_{{{\text{eq}}}} )}}{T(t)} = \frac{T( - )}{{T( - )}} = T( + )$ differs from that on the right side of Eq. (4) $T\left( {\frac{{R^{2} }}{{I_{{\text{c.m.}}} \Omega_{{\text{R}}} }}} \right)T({\mathbf{L}}_{{\text{R}}} )T({\mathbf{F}}_{{\text{s}}} ) = T( + )T( - )T( + ) = T( - )$ in contrast to a standard gyroscope with $\frac{{T({{\varvec{\uprho}}})}}{T(t)} = \frac{T( + )}{{T( - )}} = T( - )$ on the left. Furthermore, the right side of Eq. (4) does not contain **M**_eq_.

Thus, we have a unique situation: **M**_eq_ defines the spatial alignment of **L**_R_ but **M**_eq_ and **L**_R_ can be either parallel or antiparallel to one another, because the torque ${{\varvec{\uptau}}}_{{\text{R}}} = {\mathbf{r}} \times {\mathbf{F}}_{{\text{s}}}$ defining **L**_R_ is independent of **M**_eq_. Hence, the field reversal should result in the reversal of **M**_eq_ but not in that of **L**_R_ as shown in Fig. 2f. In other words, the reversal of ${\mathbf{F}}_{{{\text{appl}}}}$ will result in the reversal of the SR trajectory, while the reversal of **B** will not. Hence, the SR leads to a kind of Lorentz force: for a given ${\mathbf{F}}_{{{\text{appl}}}}$ and **B** a revolving sphere drifts to the right or to the left with ${\mathbf{v}}_{{\text{R}}} (t)$. However, the sign of the Lorentz drift can be switched by the reversal of both, **B** or ${\mathbf{F}}_{{{\text{appl}}}}$, while that of the SR-drift by the reversal of ${\mathbf{F}}_{{{\text{appl}}}}$ only. This is the consequence of the time-reversal violation.

These conclusions were checked by an experimentally letting hard magnetized NdFeB spheres to roll down an incline (see description and Movie S1–S2^8^). Initially, **M** (and the sphere) slowly precessed around ${\mathbf{T}}_{{\text{c.m.}}}$ until the **M**_eq_ corresponding to ${\mathbf{T}}_{{\text{c.m.}}} \to 0$ was reached. Then, it rotated up around **M**_eq_ and moved down an incline in agreement with the theoretical predictions. **M**_eq_ was always collinear to **L**_R_, but it was parallel or antiparallel to it depending on ${\mathbf{F}}_{{{\text{appl}}}}$. Furthermore, one can switch the sign of angular velocity ${{\varvec{\Omega}}}_{R}$ by changing the orientation of **B** with respect to the plane spanned by ${\mathbf{F}}_{{{\text{appl}}}}$ and the surface normal. The gyroscopic drift of the revolving magnet can be seen in Movie S3^8^, where the reversal of ${\mathbf{F}}_{{{\text{appl}}}}$ leads to the reversal of ${\mathbf{v}}_{{\text{R}}} (t)$. The drift direction can be described by the Eq. (4).

**Figure 3:**
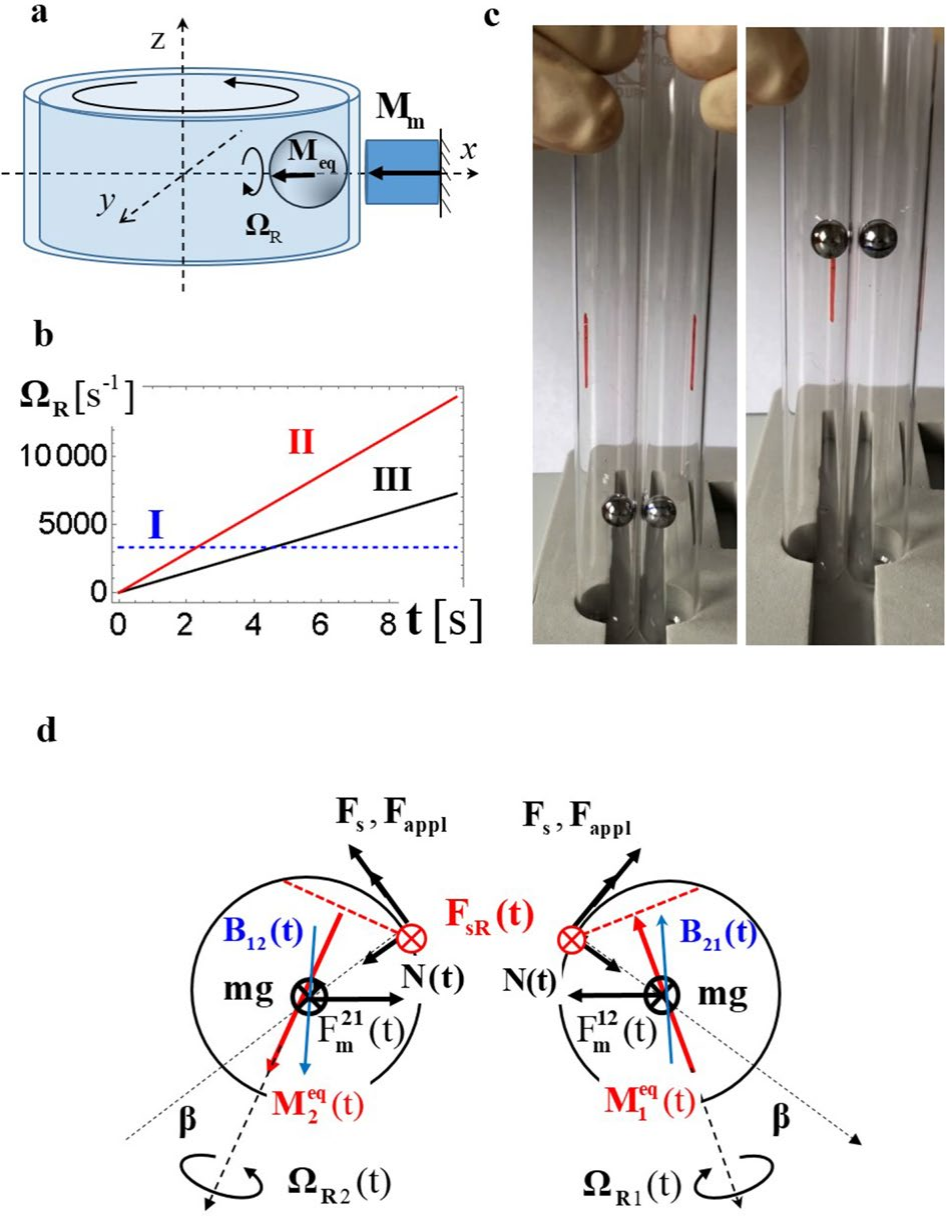
Different embodiments of the spin revolution. (**a**) Reciprocal embodiment leading to revolution of a sphere without its lateral displacement. Reprinted with permission from^11^; (**b**) Numerically calculated $\bf{\Omega }_{{\text{R}}}$ for a NiCoB sphere with $m = 3 \cdot 10^{ - 4}$ kg, $R = 3 \cdot 10^{ - 3}$ m and $M_{{\text{s}}} = 0.5\;{\text{A}} \cdot {\text{m}}^{2}$, and friction coefficient $k = 0.1$ for **I** embodiment of (**a**) with $v = 5$ m/s, **II** rolling down an incline with $\beta = \pi /10$ and $B = ( - 0.95, - 0.22,0.22)10^{ - 5}$ T; and **III** rolling down an incline with $\beta = \pi /10$ and $B = (0,1,0)10^{ - 5}$ T; (**c**) Two subsequent snap-shots of magnetic spheres (NiCoB, $m = 5 \cdot 10^{ - 4}$ kg, $R = 3 \cdot 10^{ - 3}$ m and $M_{{\text{s}}} = 0.5\;{\text{A}} \cdot {\text{m}}^{2}$) moving upwards inside of two non-magnetic tubes. (**d**) Top view of the set-up (**c**). Red arrows indicate the magnetic moments, red dashed lines show the rolling planes, red circles indicate the orientation of rolling friction. Blue arrows represent magnetic fields, black arrows show forces.

In a reciprocal version of this experiment one can fix the revolving sphere by additional magnet $\vec{M}_{{\text{m}}}$ and move the rolling surface instead of the sphere to achieve the SR (see Fig. 3a, Movie S4^8^).

In the next step we quantify the angular velocity ${{\varvec{\Omega}}}_{{\text{R}}}$ for a spontaneous rolling down an incline and a driven rolling as shown in Fig. 3a. For the rolling down an incline with ${{\varvec{\Omega}}}_{{\text{R}}} \bot {\mathbf{v}}$, an acceleration *a* can be found analytically because of the simplification ${\mathbf{v}}_{{\text{R}}} {\parallel }{\mathbf{v}}$ (see^8^, part C):

5 $$a = \frac{5}{2}\frac{{F_{{\text{s}}} }}{m} = \frac{5}{7}\;g\sin \upbeta$$

Interestingly, it depends neither on $\theta_{{{\text{eq}}}}$ nor on the mass *m*. Generally, ${\mathbf{v}}_{{\text{R}}} \ne {\mathbf{v}}$ (Fig. 2f) and can be found numerically by deriving **M**_eq_ from Eq. (2), inserting the result into Eq. (1), and solving Eqs. (1)–(4).

Figure 3b shows ${{\varvec{\Omega}}}_{{\text{R}}}$ of a NdFeB sphere in three cases: **I** corresponding to the set-up of Fig. 3a with linear velocity ${\mathbf{v}} = 5$ m/s; **II** corresponding to the rolling down an incline with $\beta = \pi /10$ and ${\mathbf{B}} = ( - 0.95, - 0.22,0.22)10^{ - 5}$ T; and **III** corresponding to the rolling down an incline with $\beta = \pi /10$ and ${\mathbf{B}} = (0,1,0)10^{ - 5}$ T. As one can see from this data ${{\varvec{\Omega}}}_{{\text{R}}}$ can be varied in a broad range by changing the applied force or inclination.

### Spin revolution effect in a system of two hard magnets

Now we switch to the SR in time-dependent fields. Let us consider two magnetic spheres, each put into a vertical non-magnetic tube. The tubes are placed close to one another and the spheres arrange themselves on internal sides of the tubes due to the magnetic attraction ${\mathbf{F}}_{{\text{m}}}^{12}$ and ${\mathbf{F}}_{{\text{m}}}^{21}$^10^ as shown in Fig. 3c,d. If the tubes are rotated about their vertical axes due to ${\mathbf{F}}_{{{\text{appl}}}}$, the spheres rotate initially together with the tubes. At a critical angle $\beta$, the sum of gravitational and magnetic forces overcomes the frictional force **F**_s_ and the spheres move upwards against intuitive expectation that they return to their initial or to somewhat lower positions in response to ${\mathbf{F}}_{{\text{m}}} + m{\mathbf{g}}$. The reason for this counterintuitive behaviour is the SR emerging at a critical angle $\beta$, when the net torque ${\mathbf{T}}_{{\text{c.m.}}}$ vanishes. According to Eq. (4) the **M**_eq_ (and spheres) should move upwards for any ${\mathbf{F}}_{{{\text{appl}}}}$. Our experiments support the expectation of emerging revolution as well as that of a lifting force defying gravity which tries to push the spheres downwards, and the magnetic interaction attracting the spheres in horizontal direction (see Fig. 3c and Movie S5^8^).

**Figure 4:**
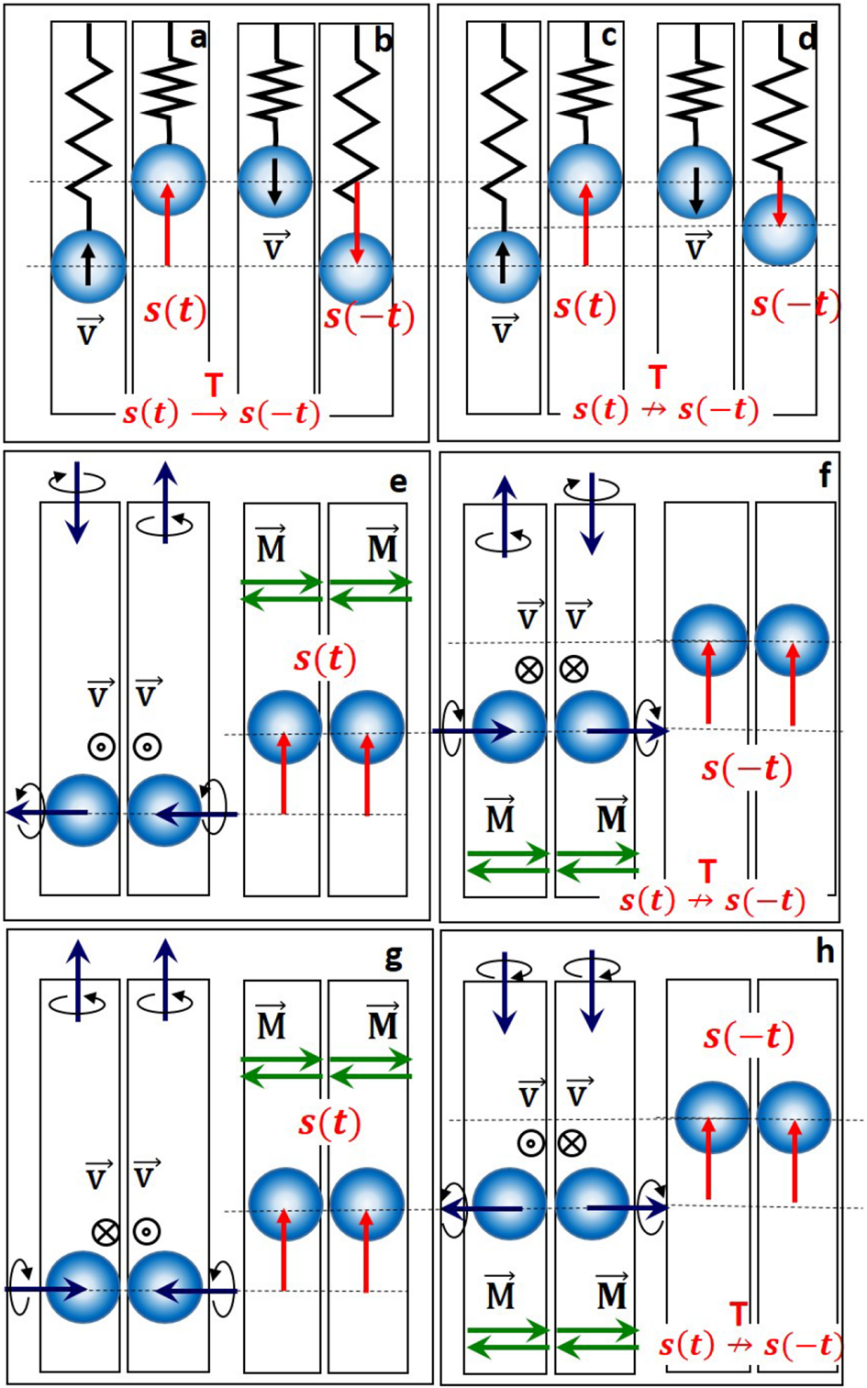
Time-reversal symmetry breaking. (**a,b**) Side-view of the forward-in-time ($s(t)$) and backward-in-time ($s( - t)$) trajectories of an ideal harmonic oscillator. Time-reversal symmetry is preserved. **(c,d)** Real harmonic oscillator with dissipation. Time-reversal symmetry is broken because $|s(t)| \ne |s( - t)|$, reversed order of events is preserved. (**e–h**) Side-view of the embodiment revealing a lifting force for different combinations of tubes’ rotations corresponding to the forward-in-time (**e,g**) and the backward-in-time motion (**f,h**). Black arrows indicate the angular momenta of the tubes and those of the spheres. Red arrows indicate trajectories. Green arrows show the allowed orientation of magnetization.

In time-reversal invariant systems, the equations of motion are invariant under the transformation $({\mathbf{q}},{\mathbf{p}},t) \mapsto^{T} ({\mathbf{q}}, - {\mathbf{p}}, - t)$ with **q** being the coordinates, **p** the momentum and *t* the time. In other words, the trajectory in reversed time should be a backward sequence of positions constituting the trajectory in forward time^16,17^. To check this, one reverses the momentum **p** and looks for the corresponding trajectory. If one reverses the rotational momentum of the tubes, the spheres will not go downwards. They will repeatedly move upwards to any tube height (Fig. 4, Movie S5^8^) breaking the time-reversal symmetry. Importantly, this symmetry breaking is neither local, like that of a Lorentz force, nor dissipation-driven. Indeed, the trajectory of a charge due to the Lorentz force becomes time-reversal invariant if the direction of magnetic field is reversed, because ${\mathbf{B}} \mapsto^{T} - {\mathbf{B}}$ (see^8^, part D and Fig. S1^8^). The only way to force the spheres moving downwards is to reverse the gravitational force. This operation is, however, forbidden as the forces are even under time-reversal (${\mathbf{F}} \mapsto^{T} {\mathbf{F}}$).

Dissipation is also a known source for the violation of time-reversal symmetry as shown in Fig. 4a–d. In this case, however, the trajectory’s length changes while the reversability of time events is not affected. In case of SR the reverse tape effect is impossible as shown in Fig. 4e–h: the time reversal results in a new trajectory. While friction is one of reasons for both phenomena: the SR and the energy dissipation, the latter is neither the reason for the SR, nor for the described time-reversal symmetry breaking. Rather, this symmetry violation stems from the emergent revolving up of the magnet and subsequent curved trajectory as explained in^8^, part E–F.

In case of tubes, the SR is achieved due to combination of magnetic attraction, friction and gravitation. It is, however, important that *mg* does not belong to the $\Pi$ plane defined by ${\mathbf{F}}_{{{\text{appl}}}}$ and **N**. If $m{\mathbf{g}} \in \Pi$, e.g. the tubes lie on a horizontal surface, the SR does not appear (see^8^, part G and Fig. S3^8^). However, already tiniest deviation from the horizontality ensures the SR. The upper limit of the lifting force can be approximated by $F_{{{\text{lift}}}} (r_{12} ) \approx F_{{\text{m}}} (r_{12} ) - kF_{{\text{m}}} (r_{12} )\cos \beta - mg$ with *k* being the friction coefficient. As the rolling friction $F_{{{\text{r}}S}}$ is tiny (0.05–0.07 for metal/plastic interfaces), $F_{{{\text{lift}}}}$ can reach significant values.

## Conclusions

To conclude, we presented a novel magnetomechanical effect consisting of rotating up a magnet and subsequent gyroscopic motion, thereby breaking time-reversal symmetry. This phenomenon offers a variety of promising applications in different fields of science and engineering including the delivery of magnetic (nano)particles. Particularly, the SR effect can be used to achieve controllable translation of objects or magnetic particles in any direction on vertical or horizontal surfaces as shown in Movie S6^8^. The advantage of this motion is the absence of direct contact between the tubes and the absence of any kind of guides increasing the weight and complexity of the system. Furthermore, the SR effect can be used for effective interconversion between rotational and translational motion that is important for linear or angle motors as shown in MovieS4^8^. The advantage of this kind of conversion is the absence of any kind of gears and versatile possibilities of switching the rotational sense. Additionally, the lifted magnets can be used for energy storage and its later harvesting using magnetic induction. An array of revolving magnets can also be utilized as information storage element. Thus, the SR effect will change our perspectives of existing magnetic phenomena and open up new technological possibilities for energy storage, energy interconversion and robotics.

## Methods

### Magnetization dynamics

To describe the time-dependence of an equilibrium magnetization orientation $\vec{M}_{{{\text{eq}}}}$, we solve numerically the set of coupled Eqs. (1)–(2) using the condition for rolling without slipping which accounts for the fact that the contact point of a sphere and a surface will be instantaneously at rest. At each time step we first solve Eq. (2) starting with a given initial magnetization orientation using the Runge–Kutta method of fourth order. When a required convergence is reached, we regard an achieved magnetization as instantaneously stable $\vec{M}_{{{\text{eq}}}} (t)$ and introduce it into the Eqs. (3) and (1). In the next step these differential equations are solved for $\vec{a}(t)$, $\vec{v}_{{\text{R}}} (t)$, and the net torque ${\vec{\mathcal{T}}}_{{\text{c.m.}}}$ using the fourth-order Runge–Kutta method. These values are then used to update the orientation of $\vec{M}_{{{\text{eq}}}} (t)$ and the position vector of the sphere. In the last step they are used as initial parameters in (2) and the entire procedure is repeated until the sphere’s c.m. and $\vec{M}(t)$ do not change with time anymore.

To describe the lifting effect, we start with a small initial rotation of the tubes by an angle $\beta$ ($\beta = \pi /10$ in Fig. 4b). The tubes will not be moved anymore, but the spheres may roll with velocity $d\beta (t)/dt$. In the next step we calculate $\vec{M}_{1,2}^{{{\text{eq}}}} (t)$ by solving two coupled equations (Eq. (2), one for each sphere) for an instantaneous $\beta (t)$. The resulting $\vec{M}_{1,2}^{{{\text{eq}}}} (t)$ are used as input parameters to find $\vec{L}_{{\text{R}}}$ and the new $\beta (t)$ and $\vec{a}$ from Eqs. (1)–(3). The procedure is repeated until $d\beta (t)/dt$ and $d\vec{M}_{1,2}^{{{\text{eq}}}} (t)/dt$ vanish.

## Supplementary Information

Supplementary Video 1.

Supplementary Video 2.

Supplementary Video 3.

Supplementary Video 4.

Supplementary Video 5.

Supplementary Video 6.

Supplementary Information 1.

## Data Availability

The codes used for this study are available from the corresponding authors on reasonable request.

## References

[1] Du L, Hasan T, Castellanos-Gomez A, Liu G-B, Yao Y, Lau CN, Sun Z (2021). Engineering symmetry breaking in 2D layered materials. Nat. Rev. Phys..

[2] Šmejkal L, Gonzáles-Hernández R, Jungwirth T, Sinova J (2020). Crystal time-reversal symmetry breaking and spontaneous Hall effect in collinear antiferromagnets. Sci. Adv..

[3] Landau LD, Lifshitz EM (1969). Mechanics.

[4] Matsuo M, Saitoh E, Maekawa S (2017). Spin-mechatronics. J. Phys. Soc. Jpn..

[5] Heims SP, Jaynes ET (1962). Theory of gyromagnetic effects and some related magnetic phenomena. Rev. Mod. Phys..

[6] Barnett SJ (1915). Magnetization by rotation. Phys. Rev..

[7] Einstein A, de Haas WJ (1915). Experimenteller Nachweis der Ampereschen Molekularströme: Experimental Proof of Ampére’s Molecular Currents. Verh. Dtsch. Phys. Ges..

[8] Supplemental Information.

[9] Vedmedenko EY, Mikuszeit N (2008). Multipolar ordering in electro- and magnetostatic coupled nanosystems. Chem. Phys. Chem..

[10] Edwards BF, Edwards JM (2017). Dynamical interactions between two uniformly magnetized spheres. Eur. J. Phys..

[11] Patent No. LU102110 Method and system involving magnetic revolution (2020).

[12] Landau LD, Lifshitz EM (1935). On the theory of the dispersion of magnetic permeability in ferromagnetic bodies. Phys. Zeits. Sowjetunion.

[13] Gilbert T (2004). A phenomenological theory of damping in ferromagnetic materials. IEEE Trans. Magn..

[14] Cross R (2015). Precession of a spinning ball rolling down an inclined plane. Phys. Teacher.

[15] Nash LM, Kleckner D, Read A, Vitelli V, Turner AM, Irvine WTM (2015). Topological mechanics of gyroscopic metamaterials. PNAS.

[16] Lamb JSW, Roberts JAG (1998). Time-reversal symmetry in dynamical systems: A survey. Physica D.

[17] Baake M, Roberts JAG (2006). The structure of reversing symmetry groups. Bull. Aust. Math. Soc..

