# Supplementary material for: Spin revolution breaks time reversal symmetry of rolling magnets
Source: Sci Rep. 2022 Aug 10;12:13608. doi: 10.1038/s41598-022-17766-z (PMC9365847; doi:10.1038/s41598-022-17766-z)
Supplement: Supplementary file 7 — Supplementary Information 1. [file 41598_2022_17766_MOESM7_ESM.pdf]

Supplementary Materials for Manuscript

Spin Revolution breaks Time Reversal Symmetry of Rolling Magnets.

Elena Y. Vedmedenko and Roland Wiesendanger

Correspondence to: vedmeden@physnet.uni-hamburg.de

This PDF file includes:

Supplementary Text
Figs. S1 to S3
Captions for Movies S1 to S6

Other Supplementary Materials for this manuscript include the following:

Movies S1 to S6

Supplementary Text

Methods:

Magnetization dynamics. To describe the time-dependence of an equilibrium magnetization orientation $\vec{M}_{\mathrm{eq}}$, we solve numerically the set of coupled equations (1)-(2) using the condition for rolling without slipping which accounts for the fact that the contact point of a sphere and a surface will be instantaneously at rest. At each time step we first solve Eq. (2) starting with a given initial magnetization orientation using the Runge-Kutta method of fourth order. When a required convergence is reached, we regard an achieved magnetization as instantaneously stable $\vec{M}_{\mathrm{eq}}(t)$ and introduce it into the Eqs. (3) and (1). In the next step these differential equations are solved for $\vec{a}(t)$, $\vec{v}_{\mathrm{R}}(t)$, and the net torque $\vec{\mathcal{T}}_{\mathrm{c.m.}}$ using the fourth-order Runge-Kutta method. These values are then used to update the orientation of $\vec{M}_{\mathrm{eq}}(t)$ and the position vector of the sphere. In the last step they are used as initial parameters in (2) and the entire procedure is repeated until the sphere's c.m. and $\vec{M}(t)$ do not change with time anymore.

To describe the lifting effect, we start with a small initial rotation of the tubes by an angle β ($\beta = \pi/10$ in Fig. 4b). The tubes will not be moved anymore, but the spheres may roll with velocity $d\beta(t)/dt$. In the next step we calculate $\vec{M}_{1,2}^{eq}(t)$ by solving two coupled equations (Eq. (2), one for each sphere) for an instantaneous $\beta(t)$. The resulting $\vec{M}_{1,2}^{eq}(t)$ are used as input parameters to find $\vec{L}_{\mathrm{R}}$ and the new $\beta(t)$ and $\vec{a}$ from Eqs. (1)-(3). The procedure is repeated until $d\beta(t)/dt$ and $d\vec{M}_{1,2}^{eq}(t)/dt$ vanish.

Code availability

The codes used for this study are available from the corresponding authors on reasonable request.

A: Rolling Torque

In the coordinate system of Fig. 1 (c) of the main manuscript, the rotational part of the motion of a rolling sphere is described by the rotational equation of motion

$$\vec{\tau}_{\mathrm{Fs}} = \vec{R} \times \vec{F}_{\mathrm{s}} = \mathbf{I}_{\mathrm{c.}m.}\varepsilon_y \tag{1}$$

with ε_y angular acceleration, $\vec{R}$ the radius, $\vec{F}_s$ the friction force, and $\mathbf{I}_{\mathrm{c.}m.} = 2/5mR^2$ the moment of inertia of a solid sphere. From the rolling condition $\varepsilon_y = a_x / R$ with a_x the linear acceleration of the center of mass and $mgsin\beta - F_s = ma_x$ (x-projection of the Newton's second law)

$$a_x = \frac{5}{7} gsin\beta \tag{2}$$

$$F_s = \frac{2}{7} mg\ sin\beta$$

and

$$\vec{\tau}_{\mathrm{Fs}} = \left(0, RF_{\mathrm{s}}, 0\right) = \left(0, \frac{2}{7} R\ mg\ sin\beta, 0\right) \tag{3}$$

B: Competition between rolling and magnetic torques

A net mechanical torque producing a change in angular momentum $\vec{L}_{\mathrm{c.}m.}$ relative to the center-of-mass reads

$$\vec{\mathcal{T}}_{\mathrm{c.}m.} = \vec{\tau}_{\mathrm{F}s}(t) + \vec{\tau}_{\mathrm{B}}(t) = \vec{R} \times \vec{F}_{\mathrm{s}} + M_{\mathrm{s}} \vec{e}_{\mathrm{M}}(t) \times \vec{B} \tag{4}$$

Here $\vec{\tau}_{Fs} = \vec{R} \times \vec{F}_s$ is a mechanical rotational torque, while $\vec{\tau}_B = M_s \vec{e}_M(t) \times \vec{B}$ the magnetic rotational torque. For the geometry of left and middle panels in Fig. 1(c) of the main manuscript only the y components of both torques do not vanish and are antiparallel. Because the vector $\vec{R}$ connecting the c.m. and a contact point is always perpendicular to $\vec{F}_s$, the minimal net torque is

$$\vec{\mathcal{T}}_{c.m.} = 0 = R \cdot F_s \sin \pi / 2 - M_s B \sin \theta_{eq} \tag{5}$$

and for small angles

$$\theta_{eq} = \frac{R \cdot F_s}{M_s B} \tag{6}$$

C: Derivation of acceleration for $\vec{v} \parallel \vec{x}$

If the direction of the velocity of the sphere's center of mass coincides with the x axis, the friction force has only one component F_{sx}. The contact point at the bottom of the sphere is instantaneously at rest. The sphere is revolving with $\vec{\Omega}_R$ about an inclined axis (see right panel of Fig. 1(c) and Fig. 2(e) of the main manuscript) and, hence, the angular moment acts at a distance r perpendicular to the x axis. Thus, one can write in a scalar form $r \cdot \Omega_R = v$ with v being the speed of the center of mass. The speed can be determined from the Newton's equation

$$m \frac{dv}{dt} = mg \sin \beta - F_{sx} \tag{7}$$

The friction force can be determined from the torque

$$F_{sx} r = \frac{2}{5} m R^2 \frac{d\Omega_R}{dt} \tag{8}$$

On the other hand, one can rewrite the torque equation describing the torque around the standard rolling axis as

$$F_{sx} R = \frac{dL_x}{dt} = \frac{2}{5} m R^2 \frac{d\Omega_R \sin \theta_{eq}}{dt} \tag{9}$$

Replacing $\Omega_R \sin \theta_{eq}$ by v / R stemming from $v = r \cdot \Omega_R = R \sin \theta_{eq} \Omega_R$, we obtain

$$F_{sx} R = \frac{2}{5} m R^2 \frac{dv}{R dt} \tag{10}$$

$$F_{sx} = \frac{2}{5} m \frac{dv}{dt} \tag{11}$$

$$a_x = \frac{5 F_{sx}}{2m} = \frac{5}{2m} \frac{2 mg \sin \beta}{7} = \frac{5 g \sin \beta}{7}$$

D: Local and global time-reversal symmetry and spatial reflection symmetry.

In classical mechanics the operation of time reversal T is defined as

$$t \overset{T}{\mapsto} -t, \ \vec{s} \overset{T}{\mapsto} \vec{s} \tag{13}$$

with t being the time and s the trajectory of an object of mass m. As a consequence the velocities and momenta (first derivatives in time) change the sign under T, while the forces and accelerations (second derivatives in time) do not ($\vec{a} \mapsto^{T} \vec{a}$, $\vec{F} \mapsto^{T} \vec{F}$). A prototypical example for time-reversal symmetry breaking is the motion of an electron in a magnetic field due to the Lorentz force $\vec{F}_L = q\vec{v} \times \vec{B}$. For the initial velocity $\vec{v}$ and the magnetic field $\vec{B}$ given in Fig. 1S the forward-in-time electron's trajectory (left) differs from its backward-in-time trajectory (top-right), if the field orientation remains unchanged. Hence, the mapping of trajectory s in Eq. 13 does not hold or, in other words, the time-reversal symmetry is *locally* broken. Globally, however, the time-reversal symmetry of this motion is preserved, because the magnetic field is odd ($\vec{B} \mapsto^{T} -\vec{B}$) under the time transformation and, hence, if the orientation of field is reversed, the forward-in-time and backward-in-time trajectories coincide (bottom-right in Fig. 1S a) and no symmetry breaking occurs.

The spatial reflection symmetry of embodiment showing lifting force is shown in Fig. 1S b. For the xy reflection plane $z \to z''$, this symmetry is preserved only if the gravitational force is also reflected. That is, from the point of view of a person in the northern hemisphere, the downwards motion of the spheres is possible only in its southern counterpart.

E: Magnetic field and magnetic attraction force

The magnetic field from a sphere 1 acting on a sphere 2 can be calculated as

$$\vec{B}_{12}(\vec{M}_1, \vec{r}_{12}) = \frac{\mu_0}{4\pi}\left(\frac{3\vec{M}_1 \cdot \vec{r}_{12}}{r^5}\vec{r}_{12} - \frac{\vec{M}_1}{r_{12}^3}\right), \tag{14}$$

where $\vec{M}_1$ is the magnetic moment of the sphere 1, while $\vec{r}_{12}$ is the distance vector between the two spheres. The attractive force acting on sphere 1 can be determined as

$$\vec{F}_1 = -\frac{\partial(\vec{M}_2 \cdot \vec{B}_{12})}{\partial \vec{r}_{12}}$$

$$\vec{F}_1 = \frac{3\mu_0}{4\pi r_{12}^5}[(\vec{M}_1 \cdot \vec{r}_{12})\vec{M}_2 + (\vec{M}_2 \cdot \vec{r}_{12})\vec{M}_1 + (\vec{M}_1 \cdot \vec{M}_2)\vec{r}_{12} - 5\frac{(\vec{M}_1 \cdot \vec{r}_{12})(\vec{M}_2 \cdot \vec{r}_{12})}{r_{12}^2}\vec{r}_{12})]$$

F: Microscopic description of the lifting force

Initially, the spheres' magnetization is oriented along the radius-vectors of the tubes (dashed black lines in Fig. 3(e) of the main manuscript). Instantaneous fields of each sphere acting on the other sphere $\vec{B}_{12}(t)$ and $\vec{B}_{21}(t)$ can be calculated exactly (9) and have small mismatch with these radius-vectors (blue arrows in Fig. 3(e), please note that all angles are exaggerated for the sake of clarity). The magnetic torque tries to rotate $\vec{M}_{1,2}(t)$ towards the instantaneous fields. The magnetic attraction force $\vec{F}_{\mathrm{m}}(t)$ (see Fig. 3(e) of the main manuscript and the chapter I of this

supplemental) acts along the line connecting the spheres' c.m. and results in tangential to the tube's surface friction $\vec{F}_s(t)$. This friction causes mechanical torque, which is antiparallel to the magnetic torque and $\vec{\mathcal{T}}_{c.m.}$ vanishes. As a result, the magnetization relaxes to an instantaneous equilibrium orientation $\vec{M}_{1,2}^{eq}(t)$ and revolves up. The noncollinearity of $\vec{M}_{eq}$ and the normal breaks the symmetry between rotation and translation and results in drift $\vec{v}_R$ defined by the intersection of the revolution plane (dashed red line in Fig. 3e)) and the tube's wall. This drifting force leads to an upward rolling of the magnets. The numerically calculated trajectory of such a motion is shown in Fig. 2S and described in the Methods section. It is a formidable task to find an analytical description of this complicated movement. The upper limit of the lifting force, however, can be approximated by $F_{lift}(r_{12}) \approx F_m(r_{12}) - kF_m(r_{12})\cos\beta - mg$ with k being the friction coefficient. As the rolling friction is tiny (0.05-0.1 for metal/plastic interfaces), the lifting force can reach significant values.

G: Other tube's geometries

In the case with two vertical tubes, the gravitational force leads to an inclination of the magnetization axis with respect to the surface's normal within the zx-plane (see Fig. 3S(a)). This deviation alone would allow the sphere to roll left or right along the tube's circumference rotating around the magnetization axis. The tube's rotation (external force $\vec{F}_{appl}$) in the xy-plane leads to the inclination of the magnetization axis with respect to the surface's normal within the xy-plane (see Fig. 3S(b)). This global inclination of the magnetization axis with respect to the surfaces' normal allows for two possible scenarios: the spheres roll up towards one another or down away from one another independent on the direction of the tube's rotation. Two other possibilities (away-upwards and down-towards are excluded by the orientation of the equilibrium magnetization axis). The away-downwards scenario leads to increasing distances between the spheres. Hence, if the magnetic attraction is stronger than the gravitation, the spheres will roll upwards decreasing magnetic potential energy and breaking the time reversal symmetry. Summarizing, the deviation of $m\vec{g}$ from to the Π plane defined by $\vec{F}_{appl}$ and $\vec{N}$ is necessary to achive the SR.

If $m\vec{g} \in \Pi$, e.g. the tubes lie on a horizontal surface, the SR does not appear, because the deviation of the magnetization axis with respect to the surface normal due to gravitation and due to tube's rotation lie in the same plane (zy-plane in Fig. 3S(c-d)). Hence, there two rolling scenarios returning the spheres back to the smallest separation distance (rolling right or left along the x-axis and towards one another) are energetically degenerate. Therefore, on average the sphere will go neither right nor left, just performing statistical movements at the origin. Already tiniest deviation from the tube's horizontality, however, is enough to achive the SR.

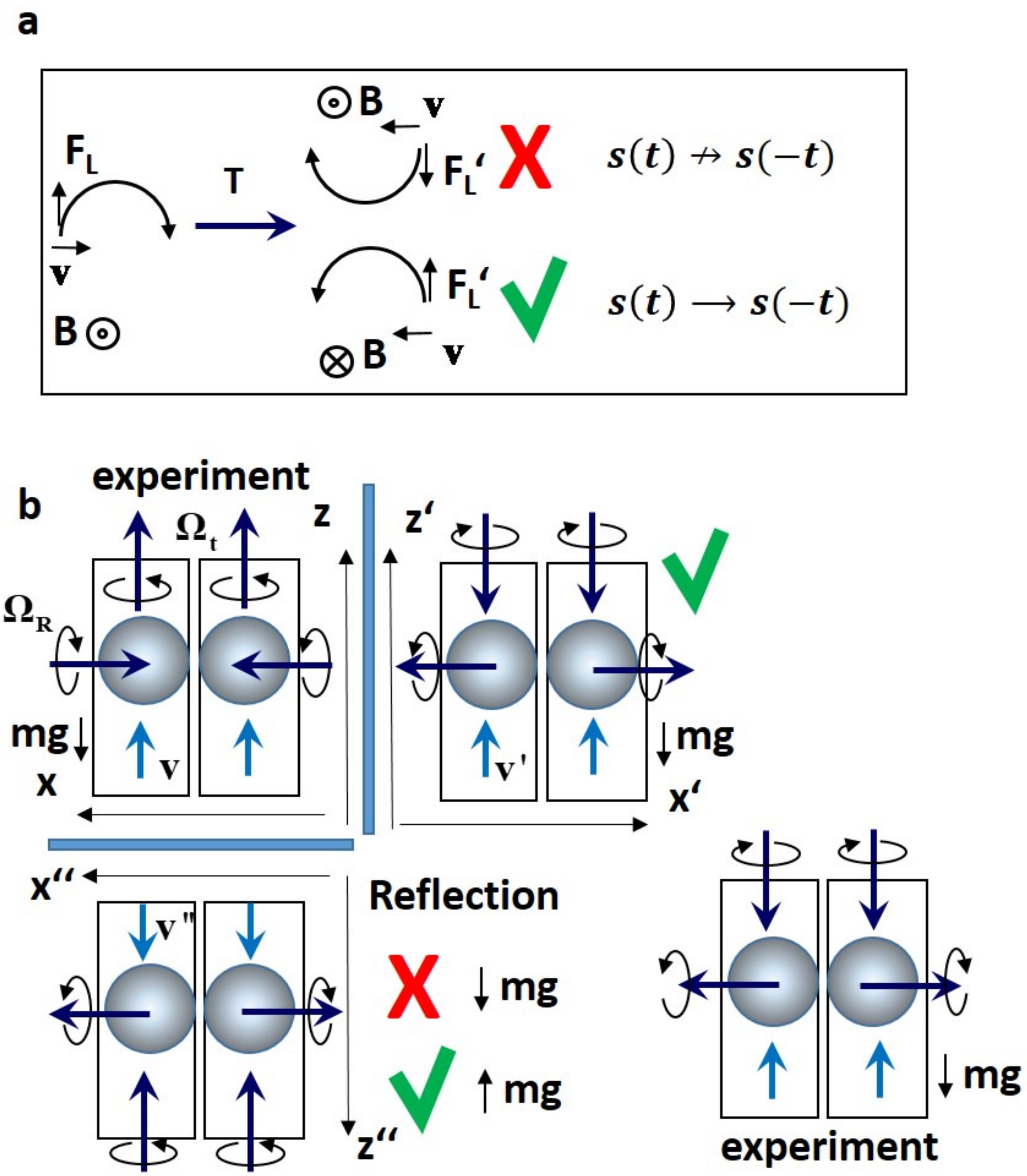

Fig. S1:
Local and global time-reversal symmetry and spatial reflection symmetry. (a) Explanation of the difference between the local and global T-symmetry on the example of Lorentz force. The red cross corresponds to the broken symmetry for non-reversed magnetic field $\vec{B}$, while the green checkmark to the preserved global symmetry. **(b)** Spatial reflection symmetry of the lifting force. The lifting force change sign only if the sign of the gravitational force is reversed.

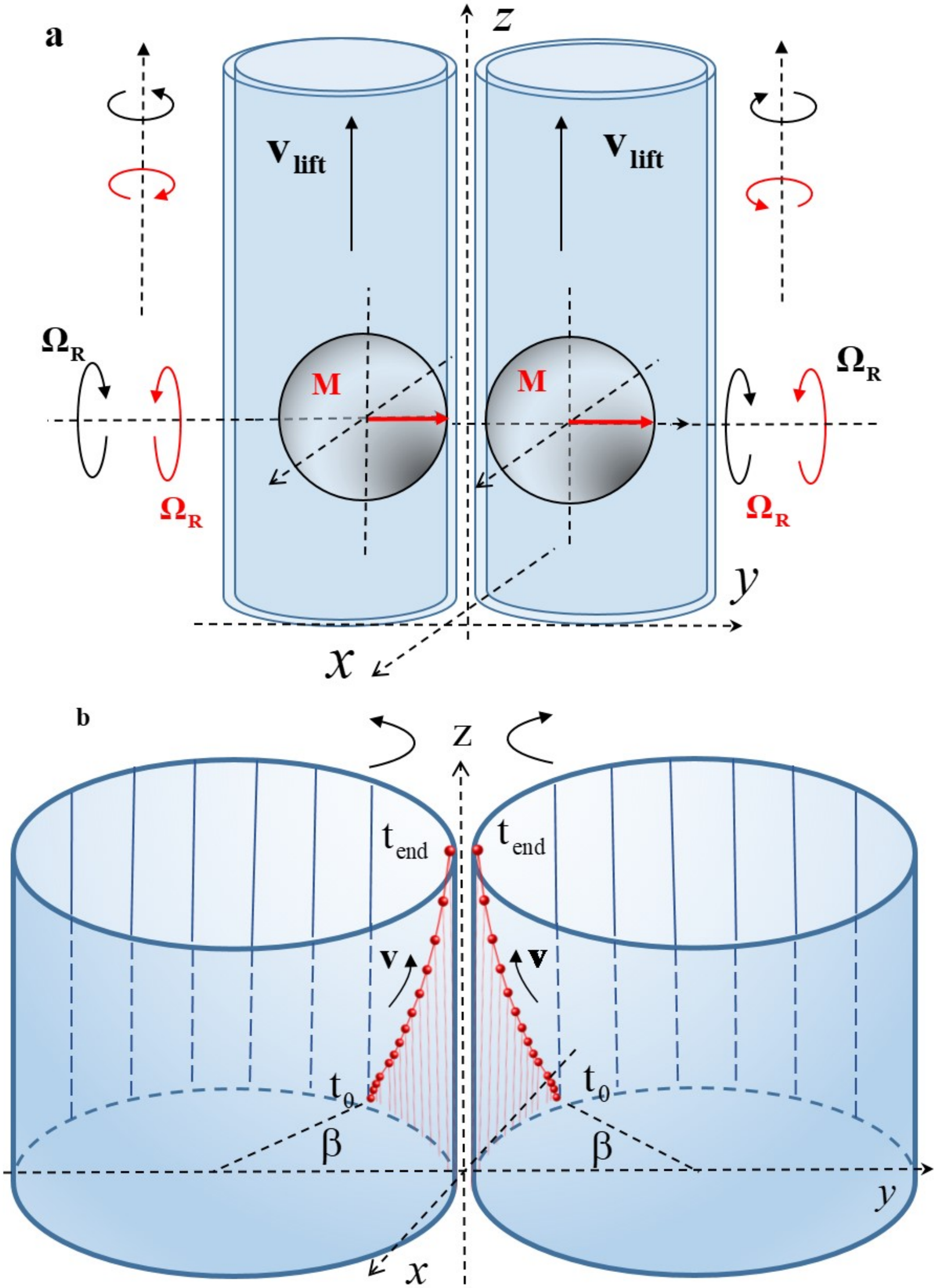

Fig. S2:
The lifting force. (a) Side-view of the embodiment revealing a lifting force (see description in text). Black and red round arrows indicate the rotation direction of the tubes and the corresponding direction of the sphere's revolution. Red line-arrows indicate $\vec{M}_{1,2}^{eq}$. **(b)** Numerically calculated trajectory of the spheres, which were initially rotated with the tubes by the azimuthal angle β. The tubes remained at this position, while the spheres were lifted revolving along the red path. In the simulations NiCoB spheres and time-steps of $dt = 10^{-7}$ s were used.

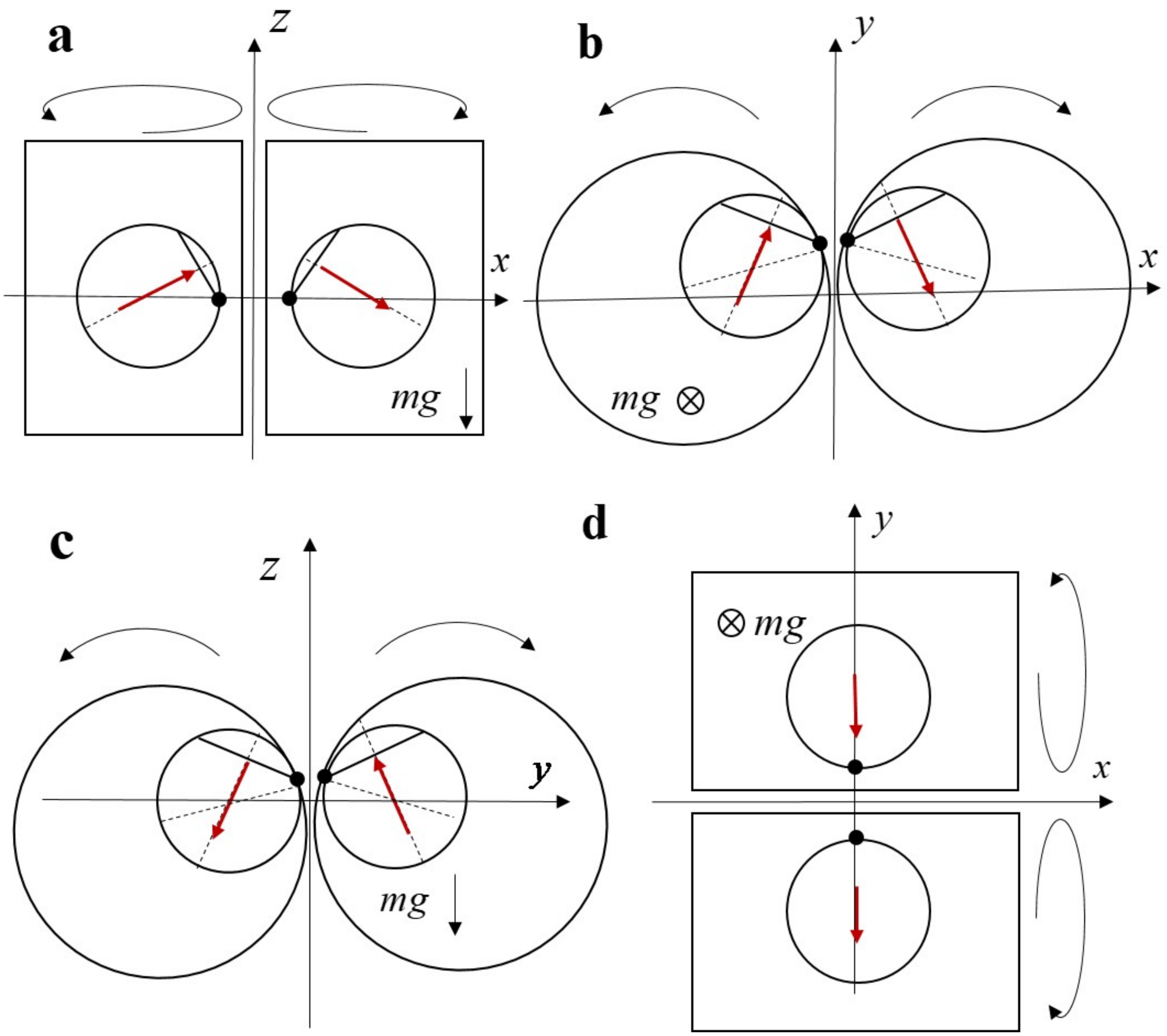

Fig. S3:
Vertical vs. horizontal tubes. Side- and top-view of two vertical non-magnetic tubes with magnetic spheres inside them **(a-b)** and horizontal nonmagnetic tubes with magnetic spheres inside **(c-d)**. Red arrows indicate the equilibrium magnetization, black solid circles the contact points, black round arrows the rotation direction of the tubes. **(a-b)** The equilibrium magnetization deviates from the Π plane defined by $\vec{F}_{appl}$ and $\vec{N}$. There is the SR and a lifting force. **(c-d)** The equilibrium magnetization lies within the Π plane defined by $\vec{F}_{appl}$ and $\vec{N}$. There is neither SR nor a lifting force.

<u>**Movie S1: The system to observe the spin revolution**</u>

The system consists of a glass or plexiglass test tube fixed on a tripod. The inclination and spatial orientation of the glass tube can be smoothly changed.

Movie S2: Spin revolution

This video shows a magnetic NiFeB sphere ($M_s \approx 0.5 \mathrm{A} \cdot m^2$, $m = 0.0003$ kg, $R = 0.003$ m) located within a test tube. Initially, its magnetization vector coincides with that of the Earth's magnetic field. The south magnetic pole of the sphere is marked by a dark cross. Inclination of the tube leads to a depart of the magnetization from its initial orientation and to a new eauilibrium magnetization orientation corresponding to the minimal net torque about the rolling axis. When the rolling torque becomes comparable with the magnetic torque, the sphere revolves up about the new equilibrium magnetization axis and rolls down the incline.

Movie S3: Change of the revolution direction and drift

If one puts a non-magnetic sphere into a test tube and lets the tube roll in the direction $\vec{v}$ coinciding with $\vec{F}_{\mathrm{appl}}$, the sphere will roll in the same direction and remain at initial position with respect to the tube due to the friction. If one replaces a non-magnetic sphere by a magnetic one (for example, $M_s \approx 0.5 \mathrm{A} \cdot m^2$, $m = 0.0003$ kg, $R = 0.003$ m), the sphere revolves up about the revolution axis and drifts in a direction that is perpendicular to the tube's velocity $\vec{d} \perp \vec{v}$. Because the angular momentum $\vec{L}_{\mathrm{R}}$ changes its sign when $\vec{v}$, $\vec{F}_{\mathrm{appl}}$ changes its direction to $-\vec{v}$, $-\vec{F}_{\mathrm{appl}}$ (see Fig. 2(f) of the main manuscript) the sphere changes the direction of drift to the opposite.

Movie S4: Flexible change of the revolution's angular velocity

Various sources of external magnetic field other than the Earth's magnetic field can be used to realize the magnetic revolution. This video gives an example of such an implementation. Here, a permanent or electro-magnet producing a field is situated underneath a non-magnetic plane, on which a magnetic sphere is positioned. Initially, the magnetic sphere (or an object of another shape) is attracted by the magnetic field and rests. In the next step, the magnetic field starts moves/changes. As a consequence, the magnet revolves-up and moves in the same direction. The strength and orientation of angular momentum depends on the strength and orientation of the field and the driving velocity. If the orientation of equilibrium magnetization reverses, the angular momentum reverses as well.

Movie S5: Lifting force

This video shows an embodiment of spin revolution, comprising two non-magnetic tubes with magnetic spheres inside them. Independently of the angular velocity of the tubes the magnetic spheres lift up the tubes.

Movie S6: Item elevator

This video shows that the lifting force can be used to lift items without using ropes, metallic rails or hydravlic elements. In order to descent the items an additional magnetic field or other means have to be applied. Without additional means the descent is not possible.
